# Dark Patterns: manipulative Designstrategien in digitalen Gesundheitsanwendungen

**DOI:** 10.1007/s00103-024-03840-6

**Published:** 2024-02-08

**Authors:** Thomas Mildner, Gian-Luca Savino, Johannes Schöning, Rainer Malaka

**Affiliations:** 1 Universität Bremen, Bibliothekstr. 5, MZH, Raum 6110, 28359 Bremen, Deutschland; 2 Universität St. Gallen, St. Gallen, Schweiz

**Keywords:** Autonomie von Nutzer:innen, Design Patterns, Datenschutz, Täuschendes Design, Mobile Anwendungen, User autonomy, Design patterns, Data protection, Deceptive design, Mobile applications

## Abstract

Digitale Gesundheitsanwendungen unterstützen Nutzer:innen unter anderem dabei, ihre physische und mentale Gesundheit durch digitale Daten besser zu verstehen, und fördern dadurch ein positives Gesundheitsverhalten. Neben den staatlich geprüften digitalen Gesundheitsanwendungen (DiGA) und digitalen Pflegeanwendungen (DiPA) besteht durch eine Vielzahl weiterer kommerzieller Gesundheitsanwendungen ein breites Angebot für Nutzer:innen. Gerade bei nicht geprüften Angeboten werden von Entwickler:innen häufig, beabsichtigt oder unbeabsichtigt, manipulative Designstrategien (Dark Patterns) verwendet, die Nutzer:innen dazu verleiten, bestimmte Entscheidungen zu treffen. Dieser Artikel bietet einen Überblick über aktuelle und weitverbreitete Dark Patterns und ordnet ein, welche Risiken von ihnen in digitalen Gesundheitsanwendungen ausgehen können.

Zukünftig sollte „Licht“ auf Dark Patterns geworfen werden, indem mehr Transparenz für Nutzer:innen geschaffen wird, Regulator:innen ein genaueres Verständnis von Dark Patterns erlangen und verstärkt auf die Umsetzung von Richtlinien geachtet wird. So können Nutzer:innen Autonomie im Umgang mit Gesundheitsanwendungen gewinnen und ihre Daten besser geschützt werden.

## Abstract (en)

Among other things, digital health applications offer users support in better understanding their physical and mental health through digital data, thereby promoting positive health behavior. In addition to state-approved digital health applications (DiGA) and digital care applications (DiPA), there is a wide array of other commercial health applications available to users. Particularly in non-approved applications, developers often deploy manipulative design strategies (dark patterns), intentionally or unintentionally, to deceive users into making specific decisions. This article provides an overview of current and widespread dark patterns and assesses the risks posed by them in digital health applications.

In the future, “light” should be shed on dark patterns by creating more transparency for users, providing regulators with a more accurate understanding of dark patterns, and paying more attention to the implementation of guidelines. Thus, users may gain autonomy using healthcare applications and their data can be better protected.

## Einleitung

Digitale Gesundheitsanwendungen (DiGA) zur Förderung der physischen oder mentalen Gesundheit erlauben es Menschen, sich im Alltag stärker mit der eigenen Gesundheit zu befassen. Oftmals steht dabei die Prävention von Erkrankungen im Vordergrund. So vereinfachen die Anwendungen beispielsweise den Zugang zu Gesundheitsinformationen oder bieten personalisierte Gesundheitspläne. Somit ermöglichen sie Nutzer:innen einen kontinuierlichen Einblick in ihren eigenen Gesundheitszustand. Dieser Artikel berücksichtigt sowohl vom deutschen Bundesinstitut für Arzneimittel und Medizinprodukte (BfArM) geprüfte und zugelassene DiGA und digitale Pflegeanwendungen (DiPA) als auch kommerzielle Produkte ohne BfArM-Anerkennung. DiGA und DiPA werden dahingehend geprüft, ob sie wissenschaftlich erwiesene Versorgungseffekte bieten. Nach der Genehmigung können sie durch medizinisches Personal verordnet werden, wobei ihre Kosten von Krankenkassen übernommen werden können. Bei Produkten ohne BfArM-Anerkennung ist dies nicht zwingend der Fall. Alle genannten Anwendungen nutzen digitale Technologien zur Erkennung, Prävention, Überwachung, Behandlung oder Linderung von Krankheiten oder Verletzungen. Der Einfachheit halber fasst dieser Artikel die drei Anwendungsformen als allgemeine digitale Gesundheitsanwendungen (ADiGA) zusammen.

Laut dem Statista Global Consumer Survey gaben im Jahr 2022 45 % der 2584 befragten deutschen Nutzer:innen an, im vergangenen Jahr ADiGA verwendet zu haben [1]. Dies untermalt das Interesse von Nutzer:innen, bei der Prävention und Förderung ihrer mentalen und physischen Gesundheit aktiv tätig zu sein. Dabei umfasst das Angebot über 41.000 Applikationen in Apples App Store [2] und über 65.000 Applikationen in Googles Play Store [3]. Der Nutzen von ADiGA findet in empirischen Studien Unterstützung: Forschungsergebnisse zeigen deutlich, dass ADiGA einen positiven Einfluss auf die Gesundheit ihrer Nutzer:innen haben können. So untersuchten Champion et al. in einer Studie, ob die Nutzung einer Mindfulness-Meditations-Anwendung positive Auswirkungen auf das psychosoziale Wohlbefinden hat, und zeigt, dass die App schon nach wenigen Wochen zu einer Steigerung der Lebenszufriedenheit, einer Reduktion des wahrgenommenen Stresses und einer Stärkung der Resilienz führte [4]. Aus technologischer Sicht ergeben sich für ADiGA zwei zentrale Herausforderungen: ein Anwendungsdesign, das Nutzer:innen in der Förderung ihrer Gesundheit unterstützt, und eine angemessene Umsetzung von IT-Sicherheit und Datenschutz [5].

Probleme entstehen vorwiegend, wenn das auf Profit ausgerichtete Geschäftsmodell eines Anbietenden und die individuellen Gesundheitsinteressen der Nutzer:innen miteinander im Konflikt stehen. Dies ist oft bei ADiGA ohne BfArM-Prüfung der Fall. Beispielsweise hat ein/e Nutzer:in einer Diät-Anwendung nach erfolgreicher Gewichtsreduktion keine Verwendung mehr für die Anwendung, weshalb er/sie dem Anbietenden als Kund:in entfällt. So besteht das Risiko für erfolgreiche Medizinprodukte, sich selbst überflüssig zu machen. Um Kund:innen stärker und langfristig an Anwendungen zu binden, werden oftmals Dark Patterns implementiert. Diese Designstrategien manipulieren oder täuschen Nutzer:innen und bewegen sie so zu ungewollten Interaktionen. Eine solche Strategie überzeugt Nutzer:innen beispielsweise durch emotional überspitzte Aussagen kostenpflichtige Abonnements abzuschließen. Häufig ist deren Kündigung zusätzlich massiv erschwert. Dabei wird die Autonomie der Nutzer:innen stark eingeschränkt. Dark Patterns basieren meist auf grafischen und/oder linguistischen Elementen, wobei tatsächliche Konsequenzen verschleiert werden, sodass Nutzer:innen über Auswirkungen unzureichend informiert sind [6, 7].

Dark Patterns und andere gezielt eingesetzte Designstrategien von ADiGA können zudem die IT-Sicherheit der Anwendungen gefährden und die Richtlinien zum Schutz personenbezogener Daten der Nutzer:innen verletzen. Das Bundesamt für Sicherheit in der Informationstechnik (BSI) veröffentlichte 2021 einen Artikel, der Mängel in 1000 populären Gesundheitsanwendungen feststellte, die im Durchschnitt lediglich 49,1 % an zuvor definierten Sicherheitsanforderungen erfüllten [8]. Dabei ist die Perspektive der IT-Sicherheit für digitale Gesundheitsanwendungen besonders relevant. Denn Daten, die in diesem Kontext entstehen, sind hoch sensibel, da sie kritische, persönliche Einblicke in die Gesundheit von Individuen zulassen. Perakslis und Coravos haben 2019 vorgeschlagen, Gesundheitsdaten als „digitale Proben“ zu betrachten und diese mit der gleichen Sorgfalt wie Blut oder anderen physischen Proben zu behandeln [9].

Vor diesem Hintergrund fokussiert sich dieser Artikel insbesondere auf Dark Patterns, die unter anderem als Ursache von IT-Sicherheitsrisiken auftreten, indem sie Nutzer:innen dazu verleiten, unbedacht personenbezogene Daten preiszugeben oder Zustimmungen zu riskanten Datenverarbeitungspraktiken zu geben, welche die Integrität und Vertraulichkeit ihrer Gesundheitsdaten gefährden können. Der Artikel ist in 3 wesentliche Abschnitte unterteilt: Zunächst wird das Konzept von ADiGA erörtert und ein genereller Überblick über ihre Eigenschaften und damit verbundene positive Möglichkeiten gegeben. Anschließend werden Dark Patterns und ihre Ursprünge im Zusammenhang mit ADiGA aufgezeigt. Zum Schluss werden effektive Ansätze vorgestellt, wie der Wirkungsraum von Dark Patterns begrenzt werden kann und somit Nutzer:innen vor entsprechenden Risiken bewahrt werden können. Ein besonderer Fokus liegt dabei auf dem von uns entwickelten „KLAR“-Konzept, das vier Handlungsaufrufe umfasst: K (klare Nutzeroberflächen und Informationen), L (Licht auf Dark Patterns werfen und diese verstehen), A (aktiver Schutz der Gesundheitsdaten von Nutzer:innen) und R (Rechenschaftspflicht der Plattformbetreiber:innen und Entwickler:innen). Dieser Ansatz zielt darauf ab, die Nutzer:innenautonomie zu stärken, Dark Patterns und ihre Konsequenzen sichtbar zu machen, die Umsetzung von Datenschutzrichtlinien zu intensivieren und die Verantwortung der Anbietenden und Entwickler:innen zu betonen.

## Digitale Gesundheitsanwendungen

ADiGA sind mobile Anwendungen, verfügbar auf Smartphones, Wearables (tragbare Technologien, wie z. B. Fitnessbänder und Smartwatches) und Tablets. Sie werden speziell dafür entwickelt, die Gesundheit ihrer Nutzer:innen zu fördern. Allgemein ist der Zugang zu ADiGA durch die weitverbreitete Verfügbarkeit von Smartphones und verwandten Technologien, wie zuvor genannte Geräteklassen, verhältnismäßig einfach und das Angebot dementsprechend vielfältig. Aufgrund der Diversität im Angebot gibt es in der Literatur allerdings keine einheitliche Definition, die Apps dieser Kategorie beschreibt [10]. Aus diesem Grund bezieht sich dieser Artikel auf alle mobilen Anwendungen, welche es ihren Nutzer:innen ermöglichen, Zugang zu ihren eigenen mentalen und physischen Gesundheitsdaten zu erlangen, um dadurch ihr generelles Wohlempfinden positiv zu beeinflussen. Beispielsweise richten sich einige Apps an die physische Gesundheit und nutzen Daten zur körperlichen Leistungsfähigkeit und der eigenen Ernährung, während andere die mentale Gesundheit verbessern wollen und z. B. Achtsamkeit fördern in Bezug auf persönliche körperliche und geistige Bedürfnisse.

Die Funktionalität von ADiGA wird vor allem durch eine Vielzahl von Sensoren in Smartphones ermöglicht. Vom Schrittzählen über das Messen des Pulses zum Aufzeichnen von Blutsauerstoff – moderne Geräte besitzen spezielle Sensorik in meist hoher Qualität und sind in der Lage, zentrale Gesundheitsdaten aufzuzeichnen. Verschiedene Applikationen können diese gezielt auslesen, um Nutzer:innen tiefere Einblicke in ihren Gesundheitszustand zu gewähren und dabei kontextbezogene Rückmeldungen zu geben. ADiGA bieten aber nicht nur Nutzer:innen große Vorteile, indem sie digitale Gesundheitsdaten leicht und verständlich darstellen [11]. Auch Ärzt:innen können ihre Patient:innen durch die Verwendung von ADiGA individuell unterstützen und auf deren Bedürfnisse eingehen [12]. Um den größtmöglichen positiven Effekt zu erzielen, ist ein häufiges Ziel von ADiGA, das Bewusstsein und Verhalten ihrer Nutzer:innen zu beeinflussen und langfristig zu ändern [13]. Dafür können Strategien wie Nudges (Anstupser) oder Persuasive Design (überzeugende Gestaltung) eingesetzt werden.

## Nudging und Persuasive Design

Damit Nutzer:innen tatsächlich gesünder und bewusster leben können, müssen viele ihre Verhaltensweisen ändern. Dazu gehören z. B. Ernährungsumstellungen, (mehr) Bewegung im Alltag oder Zeit, um mentale Achtsamkeit zu üben. Für viele ist dies kein einfacher Weg, weshalb App-Entwickler:innen auf Designstrategien zurückgreifen, um Nutzer:innen zu gesünderen Handlungen oder Tätigkeiten anzuregen.

Sogenannte Nudges (Anstupser) können durch gezielt gestaltete Benutzungsoberflächen genau dies bewirken; sie greifen dabei allerdings in die Entscheidungsautonomie der Nutzer:innen ein. Das Konzept der Nudges wurde erstmals 2008 von Thaler und Sunstein eingeführt. Sie definieren einen Nudge als subtile Intervention, die gezielt in den Entscheidungsraum eingreift [14]. Ein prominentes Beispiel aus dem Gesundheitswesen findet sich in den verschiedenen Ansätzen, Menschen zur Organspende zu bewegen [15]. Einige Regierungen nutzen dafür Nudging effektiv, indem sie die Zustimmung zur Organspende gesetzlich als Standard festlegen und Bürger:innen aktiv widersprechen müssen, um nicht als Spender:innen zu gelten. Dieser subtile, aber wirkungsvolle Ansatz, der das Prozedere der Beantragung eines Organspendeausweises umkehrt, hat in Ländern wie Österreich und Belgien bereits beeindruckende Ergebnisse erzeugt. Seit der Implementierung dieser Strategie im Jahr 2003 sind über 98 % der Bevölkerung in diesen Ländern Organspender:innen [16]. In Deutschland, wo die Organspende eine aktive Entscheidung ist, waren es im selben Jahr lediglich 12 %. Im Jahr 2022 gab die Bundeszentrale für gesundheitliche Aufklärung (BZgA) an, dass der Anteil auf 40 % Organspender:innen innerhalb der Bevölkerung angestiegen sei. Zwar zeigten 80 % aller Befragten Bereitwilligkeit zur Organspende, jedoch besaßen viele keinen offiziellen Organspendeausweis [17].

Im Bereich Web- und App-Entwicklung bedienen sich Entwickler:innen häufig ähnlicher Strategien aus der Schule des Persuasive Design [18]. Solche Strategien sind darauf ausgelegt, vor allem grafische Elemente gezielt einzusetzen, um Nutzer:innen effizient durch eine Interaktionssequenz zu einem Ziel zu führen. Oft bezieht sich dies auf Kaufentscheidungen, welche schnell abgewickelt werden sollen. Die Designelemente können Nutzer:innen helfen einfacher durch komplexe Bedienungsoberflächen zu navigieren. Allerdings können die Dienstleistenden damit auch andere Ziele verfolgen, die für Nutzer:innen schwer erkennbar sind. Designer:innen und Entwickler:innen können hier auf ein großes Repertoire von etablierten Gestaltungsmustern (Design Patterns) zurückgreifen, zu denen auch die problematischen Dark Patterns gehören. Design Patterns bieten effiziente, wiederverwendbare Lösungen zu Designproblemen und beruhen auf der von Alexander, Ishikawa und Silverstein bereits 1977 vorgestellten „Pattern Language“ [19]. In diesem Zusammenhang wurde unter Wissenschaftler:innen vermehrt festgestellt, dass einige dieser Strategien, anders als Nudges, die Autonomie der Nutzer:innen stark einschränken. Verschiedene Forschungsgruppen im letzten Jahrzehnt haben eine Vielzahl individueller und kontextspezifischer unethischer Designs typologisiert, die von Gray et al. in sechs übergeordneten Dark Patterns [20] zusammengefasst wurden.

## Dark Patterns

Im vergangenen Jahrzehnt haben Wissenschaftler:innen über 80 Dark Patterns in verschiedenen Anwendungsgebieten identifiziert und beschrieben [21]. In diesem Abschnitt werden die sechs von Gray et al. [7, 20] identifizierten übergeordneten Dark-Patterns-Strategien vorgestellt und deren Wirkung jeweils anhand eines Beispiels illustriert, das sich an einer aktuellen, vielgenutzten Gesundheitsanwendung orientiert.

### Nagging

**Figure 1:**
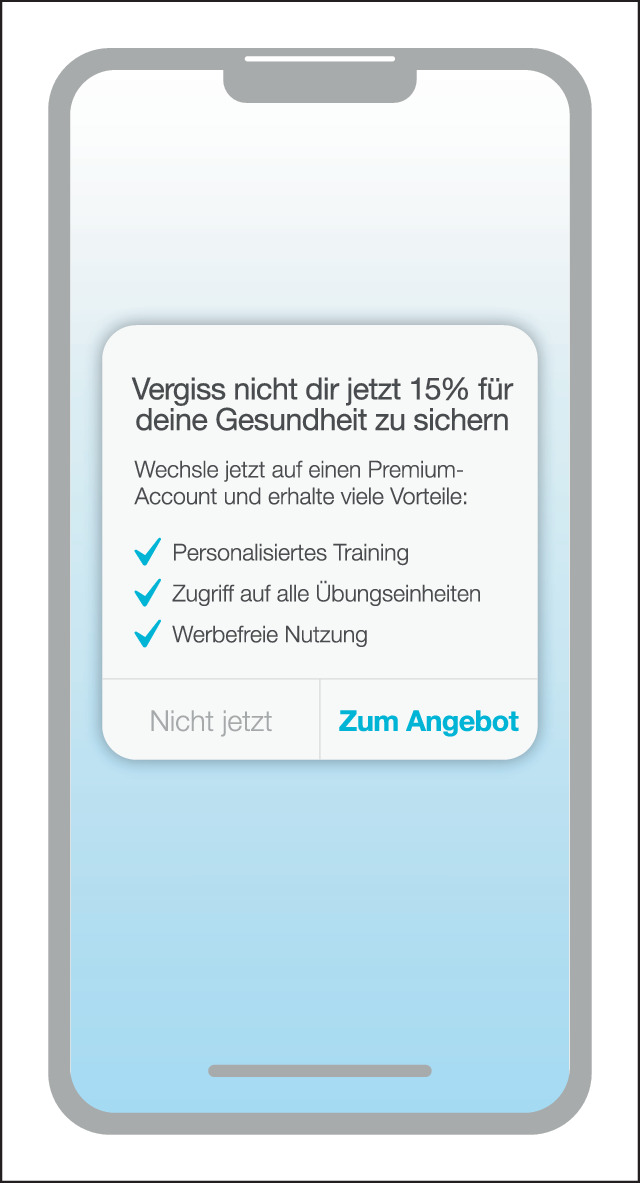

Nagging (Nörgelei) beschreibt eine Änderung der erwarteten Funktionalität, die über eine oder mehrere Interaktionen hinweg bestehen kann. Wie Abb. 1 illustriert, äußert sich Nagging häufig als wiederholte Unterbrechung während der normalen Nutzung einer App. Nagging kann Aufforderungen oder Werbung für kostenpflichtige Funktionen beinhalten, welche von Nutzer:innen nicht endgültig abbestellt werden können. Häufig stören diese Pop-ups bei der Nutzung einer App, indem sie Oberflächen verdecken, durch akustische Hinweise irritieren oder anderweitig Interaktionen behindern [7].

### Obstruction

**Figure 2:**
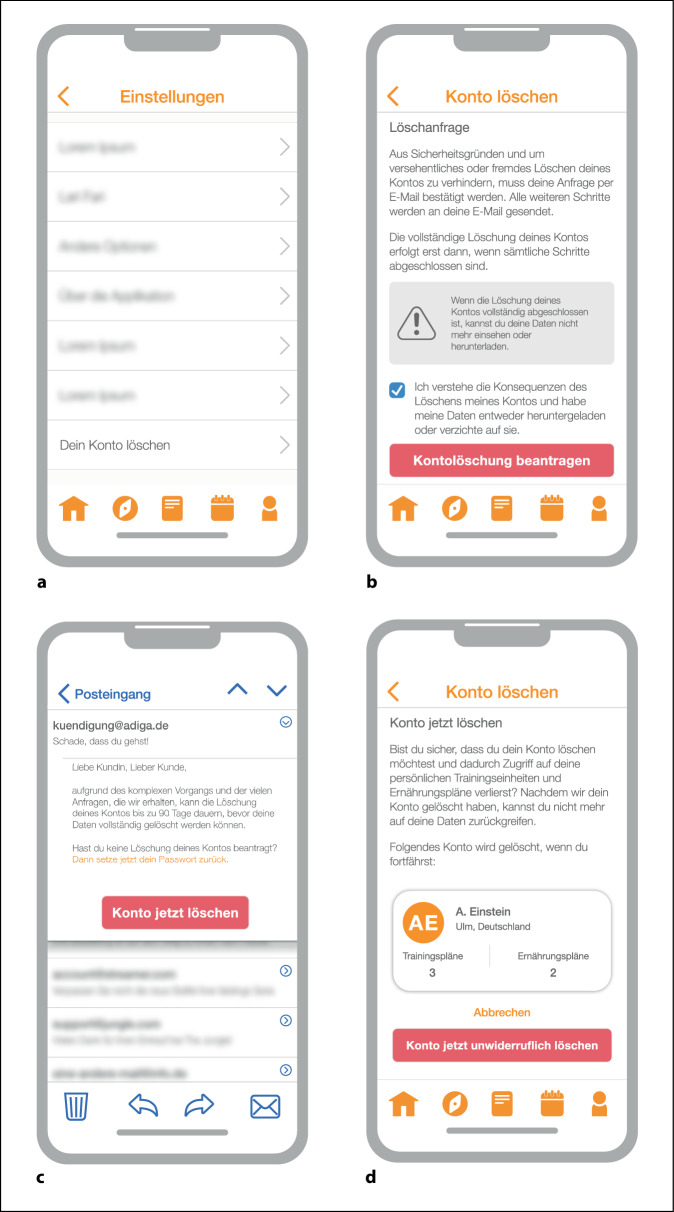

Obstruction (Behinderung) beschreibt das Behindern eines Arbeitsablaufs, indem eine Interaktion schwieriger als notwendig gestaltet wird. Obstruction äußert sich häufig als großes Hindernis für eine spezielle Aufgabe, die der/die Nutzer:in möglicherweise ausführen möchte [7]. Dies geschieht mit der Absicht, Nutzer:innen zu entmutigen eine Aktion durchzuführen, wie etwa die Löschung eines Kundenkontos (Abb. 2).

### Sneaking

**Figure 3:**
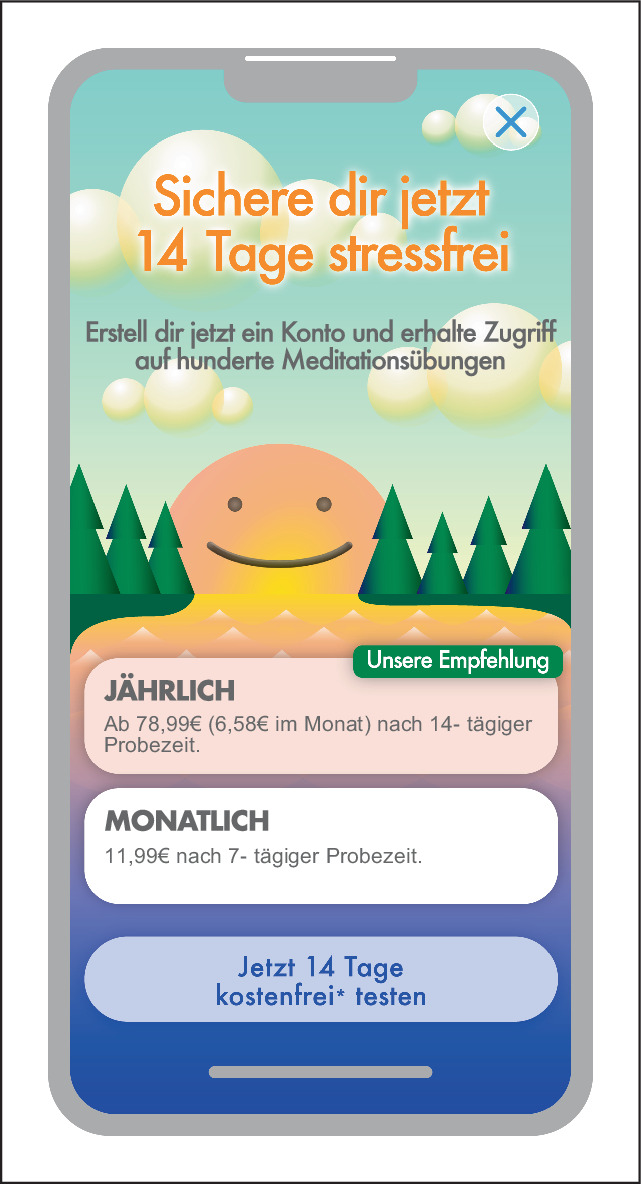

Wie Abb. 3 zeigt, beschreibt Sneaking (Heimlichtuerei) den Versuch, relevante Informationen zu verstecken, zu tarnen oder deren Offenlegung zu verzögern. Nutzer:innen werden zu einer Aktion verleitet, indem Vorteile präsentiert werden. Dabei sind die genauen Bedingungen schwer einsehbar, was verhindert, das Nutzer:innen Konsequenzen rechtzeitig reflektieren können. Mittels Sneaking können beispielsweise zusätzliche Kosten oder unerwünschte Auswirkungen einer bestimmten Aktion verschleiert werden [7].

### Interface Interference

**Figure 4:**
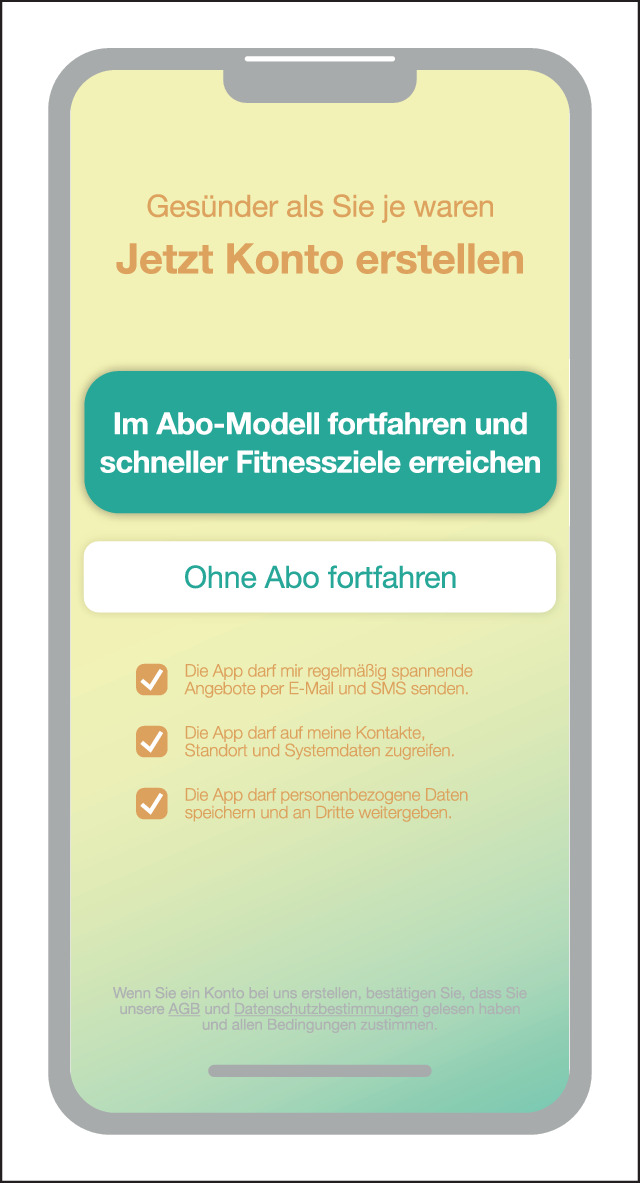

Interface Interference (etwa Störung von Oberflächen) beschreibt Manipulationen der Benutzungsoberfläche, die dazu führen sollen, dass bestimmte Aktionen gegenüber anderen bevorzugt werden, wodurch die Nutzer:innen verwirrt werden oder die Entdeckbarkeit wichtiger Handlungsmöglichkeiten eingeschränkt wird. Interface Interference, illustriert in Abb. 4, äußert sich in zahlreichen individuellen visuellen und interaktiven Täuschungen, wie zum Beispiel dem Hervorheben des Cookies-akzeptieren-Buttons durch eine prominente Farbe [7].

### Forced Action

**Figure 5:**
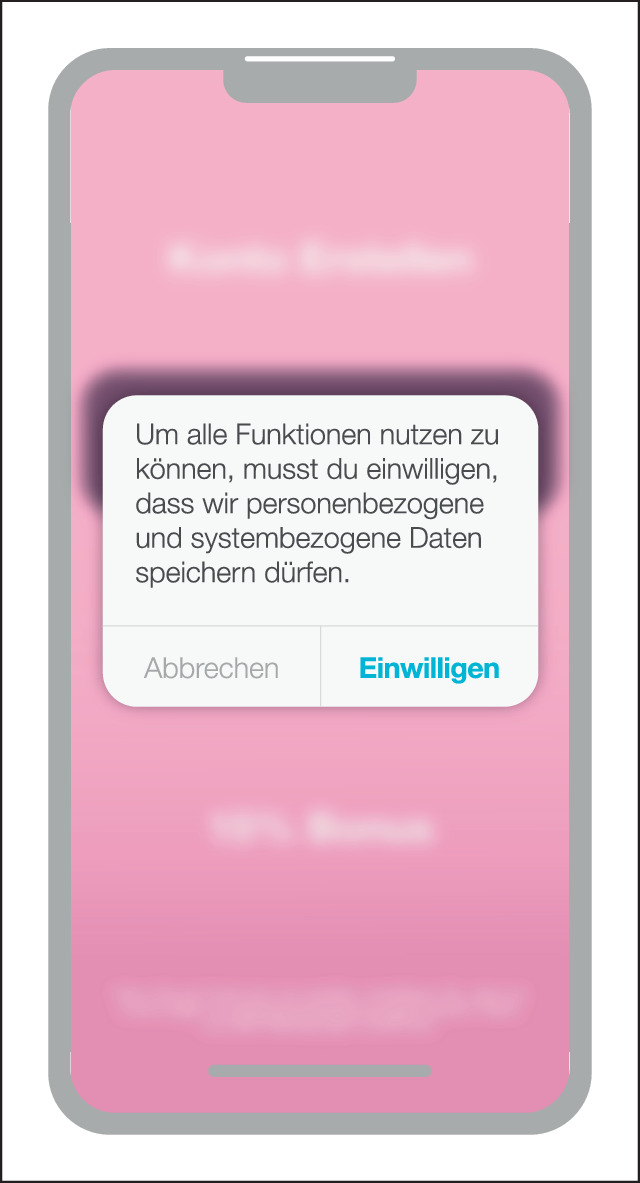

Forced Action (erzwungene Handlung) beschreibt Situationen, in denen Nutzer:innen eine spezifische Aktion ausführen müssen, um auf bestimmte Funktionalitäten (weiterhin) zugreifen zu können (Abb. 5). Diese Aktion kann sich als erforderlicher Schritt zur Vervollständigung eines Prozesses erweisen. Sie kann als eine Option getarnt sein, von der die Nutzer:innen stark profitieren würden [7].

### Social Engineering

**Figure 6:**
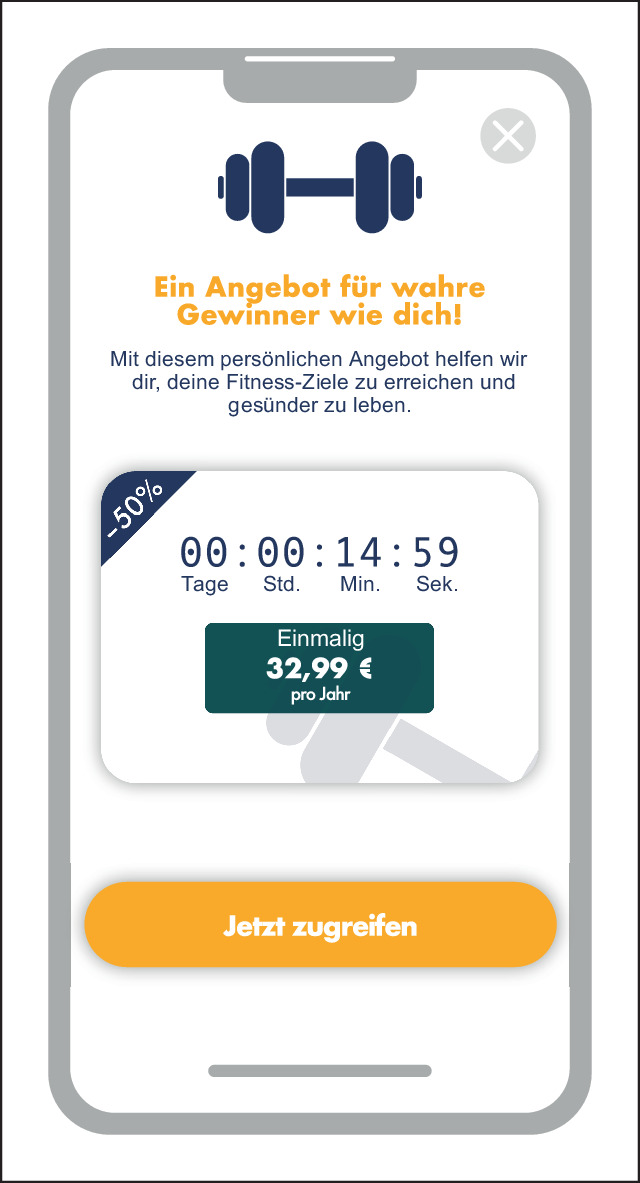

Social Engineering bezeichnet in diesem Kontext eine Strategie, die sozialpsychologische Mechanismen zur Beeinflussung und Steuerung des Nutzungsverhaltens verwendet. Diese Mechanismen können das Bedürfnis nach Zugehörigkeit, die Angst vor Verlust oder die Reaktion auf Dringlichkeit und Knappheit beinhalten. Durch diese Mechanismen wird ein Umfeld erzeugt, das die Nutzer:innen zu gewünschten Interaktionen leitet, wie Abb. 6 veranschaulicht [20, 22].

## Dark Patterns in Gesundheitsanwendungen

Wie sich Dark Patterns negativ auf Gesundheitsanwendungen auswirken können, zeigen die zuvor dargestellten Beispiele. Solche und ähnliche Dark Patterns sind in verschiedenen Anwendungskategorien zu finden. Im Bereich Ernährungsanwendungen untersuchte eine Studie von Eikey [23] die ungewollten negativen Auswirkungen von Ernährungs- und Fitness-Apps auf Frauen mit Essstörungen im Universitäts- und Hochschulumfeld. Die Ergebnisse zeigen, dass diese Apps Symptome auslösen und verschlimmern können, indem sie sich stark auf Quantifizierung konzentrieren, übermäßige Nutzung fördern und fehlleitende Rückmeldungen geben. Nach Erkennung von ungesundem Verhalten erhielten Nutzer:innen beispielsweise negativ formulierte Warnungen (Social Engineering), welche zu übertriebenen und noch ungesünderen Reaktionen führten. Die Studie betont die Notwendigkeit eines grundlegenden Wandels in der Art und Weise, wie Diät- und Fitness-Apps Gesundheit fördern, wobei die psychische Gesundheit mehr in den Vordergrund gestellt werden sollte.

Manche dieser negativen Effekte basieren auf der grundlegenden Motivation, Nutzer:innen länger auf Plattformen zu halten oder sie immer wieder dorthin zurück zu navigieren, um beispielsweise mehr Werbezeit und dadurch höhere Einnahmen zu erzielen. Im Bereich sozialer Netzwerke liegen an dieser Stelle bereits empirisch erforschte Dark Patterns vor [21], welche vor allem unter die Strategien Obstruction und Social Engineering fallen würden.

Zwar gibt es bisher keine vergleichbaren Ergebnisse aus dem Bereich Gesundheitsanwendungen, jedoch ist bekannt, dass auch einige Apps dieser Kategorie Dark Patterns implementieren, wie die obigen Beispiele zeigen. Soziale Elemente für die Interaktion mit anderen Nutzer:innen bieten hier Anwendungsmöglichkeiten. Prinzipiell zielen die Methoden darauf ab, Nutzer:innen einerseits durch unterhaltende oder spielerische Elemente aktiv an die jeweilige Plattform zu binden und andererseits selbstständige Entscheidungen einzuschränken, indem diese durch Design und Interaktionen im Sinne der Betreibenden beeinflusst werden. An dieser Stelle werden häufig Forced Actions genutzt, die Nutzer:innen zu bestimmten Handlungen zwingen, bevor die tatsächlich gewünschten Aktionen ausgeführt werden können.

Dass ein Dark Pattern selten alleine kommt, wenn Nutzer:innen bestimmte Aktionen besonders erschwert werden sollen, zeigt eine weitere Studie von Mildner et al. [24]. Beispielsweise können Interface Interference, Obstruction und Social Engineering das Löschen eines Kundenkontos in vielen Anwendungen gemeinsam erschweren: Zunächst könnte die Funktion zum Löschen in der Anwendungsoberfläche durch andere Optionen verdeckt bzw. in Menüs versteckt sein (Interface Interference). Während der Durchführung könnten emotionale Texte bei Nutzer:innen Schuldgefühle, Scham oder Angst auslösen, indem sie z. B. auf nicht mehr verfügbare Funktionen aufmerksam machen und drastische Konsequenzen ankündigen (Social Engineering). Der gesamte Löschprozess könnte sich über diverse Schritte abwickeln (Obstruction) und dabei wieder und wieder visuell und textlich Entscheidungen beeinflussen.

Allgemein suggerieren Dark Patterns böswillige Absichten von Anbieter:innen. Darum ist es wichtig, darauf aufmerksam zu machen, dass anbieterseitig auch versehentlich Probleme implementiert werden können, die Nutzer:innen schaden können. Ein Beispiel aus dem sozialen Netzwerk Reddit verdeutlicht dies: Hier beschwert sich eine Person darüber, dass ihre Apple Watch sie durch ständige Benachrichtigungen (Nagging Dark Patterns) vehement an ihre Fitnessziele erinnerte und ihr damit ein schlechtes Gewissen machte, obwohl die besagte Person in derselben Zeit an COVID-19 erkrankt war [25]. Solche gut gemeinten, motivierenden Nudges, wie sie ferner auch im Sneaking Dark Pattern beschrieben sind, werden bei Fitness-Apps immer wieder dazu verwendet, Nutzer:innen an ihre aktuellen Trainingsziele zu erinnern. Ein anderes Beispiel beschreibt, wie das Ziel von 10.000 Schritten täglich an Tagen, an denen die Luftqualität durch Luftverschmutzungen so gering ist, dass Wetter- und Gesundheitsexpert:innen empfehlen, das Haus nicht zu verlassen, negative gesundheitliche Konsequenzen haben kann [26].

Es sind hier weniger DiGA oder DiPA, sondern andere kommerzielle Anwendungen ohne BfArM-Zulassung, welche durch geringere Regulierung die meisten Risiken mit sich bringen. Eine BfArM-Zulassung hat zwar keinen direkten Einfluss auf die Verwendung von Dark Patterns. Dennoch unterscheiden sich die Anreize und damit die gewählten Design Patterns aufgrund der unterschiedlichen Finanzierungen. Gerade sogenannte Freemium-Anwendungen, d. h. zunächst kostenfreie, hochwertige Apps, die durch In-App-Käufe, Werbung und den Verkauf von Daten an Dritte Einnahmen generieren, sollten hier betrachtet werden. Denn diese Apps besitzen, bedingt durch ihre Geschäftsmodelle, Anreize, Dark Patterns einzusetzen, die Nutzer:innen auf ihren Plattformen halten und deren Handlungen steuern.

## Gesundheitsanwendungen für die Zukunft

Mittlerweile sind neben Wissenschaftler:innen aus dem Bereich Mensch-Computer-Interaktion (Human-Computer Interaction – HCI) auch Rechtswissenschaftler:innen auf die durch Dark Patterns verursachten Probleme, vor allem in Bezug auf personenbezogene Daten, aufmerksam geworden. Während mithilfe der Datenschutz-Grundverordnung (DSGVO; [27]) bereits 2016 ein großer Schritt innerhalb der Europäischen Union (EU) gemacht wurde, um Menschen im Netz mehr Schutz zu bieten, besitzt die EU durch den seit 2022 gültigen Digital Services Act (DSA; [28]) erstmals gezielt gegen Dark Patterns gerichtete Regulationen. Allerdings sollte an dieser Stelle erwähnt werden, dass Verordnungen und Regulationen zwar notwendig sind, um manipulative Praktiken in die Schranken zu weisen, Unternehmen diese aber immer wieder durch kreative Interpretationen umgehen, um weiterhin das Verhalten ihrer Nutzer:innen zu kontrollieren. Anhand verschiedener Interpretationen bei der Implementation von Cookie-consent-Bannern wird dies schnell ersichtlich: Empirische Studien haben wiederholt ergeben, dass ein Großteil der indexierten Webseiten Nutzer:innen unzureichende Auswahlmöglichkeiten bieten und dabei nicht konform mit DSGVO-Richtlinien sind [29]. Aber selbst solche Banner, die technisch konform sind, nutzen Dark Patterns, um persönliche Daten an Dritte weitergeben zu können [30]. Diese Umstände zeigen deutlich auf, dass hier Nachbesserungen notwendig sind. Umso mehr, wenn es um den Umgang mit sensiblen Gesundheitsdaten geht.

Neben mangelnden Richtlinien und Regulierungen liegt also ein großes Problem in der Umsetzung und konsequenten Überprüfung bereits vorhandener Vorgaben. Um effektiv gegen unethische Praktiken vorzugehen, werden verschiedene parallele Ansätze benötigt. Diese sollten Nutzer:innen ausreichend Autonomie bieten, aber auch das Ziel haben, Entwickler:innen ausreichend zu schulen [31], um unethische Designs zu vermeiden. Ein zentrales Problem liegt hier nicht nur in den immer neuen Dark Patterns, sondern auch in der häufig achtlosen Übernahme von zwar bewährten, aber eben schädigenden Designstrategien. Der kontemporäre Diskurs um Dark Patterns betrifft daher nicht ausschließlich böswillige Intentionen, sondern bezieht sich mit der Bezeichnung „dark“ vor allem auf versteckte Konsequenzen aus Sicht der Nutzer:innen.

Mit Rückblick auf die bestehenden empirischen Studien und derzeitige Regulierungen stellt dieser Artikel erstmals „KLAR“ vor: vier Handlungsaufrufe, um Nutzer:innen in Zukunft eine sichere Nutzung von Gesundheitsanwendungen zu ermöglichen:

*K* Klare Nutzeroberflächen entwickeln und Informationen bereitstellen, die über Handlungskonsequenzen eindeutig aufklären.

*L* Licht auf Dark Patterns werfen und diese verstehen, um effektive Präventivmaßnahmen zu etablieren.

*A* Aktiven Schutz der gesundheits- und personenbezogenen Daten von Nutzer:innen durch Entwicklung und Durchsetzung von strengeren Regularien gewährleisten.

*R* Rechenschaftspflicht für Plattformbetreiber:innen und Entwickler:innen im Falle von Missachtung geltender Regularien einführen.

Das Akronym „KLAR“ betont die Bedeutung der Transparenz in Bezug auf die Stärkung der Nutzer:innen-Autonomie im Umgang mit Gesundheitsanwendungen, der Kenntniserweiterung bei Regulator:innen zu Dark Patterns, einer aktiven Umsetzung von Richtlinien, um die Privatsphäre von Nutzer:innen zu schützen und um Plattformbetreiber:innen und Entwickler:innen zur Rechenschaft zu ziehen.

## Fazit

Digitale Gesundheitsanwendungen bieten zur Erhaltung und Förderung von Gesundheit neue Möglichkeiten, erfordern jedoch auch einen verantwortungsvollen Umgang, um ihre Wirksamkeit und Sicherheit zu gewährleisten. Unethische Strategien können positive Effekte schmälern oder Nutzer:innen sogar schaden. Zukünftig sollte „Licht“ auf Dark Patterns geworfen werden, indem mehr Transparenz für Nutzer:innen geschaffen wird, Regulator:innen ein genaueres Verständnis von Dark Patterns erlangen und verstärkt auf die Umsetzung von Richtlinien geachtet wird. So können Nutzer:innen Autonomie im Umgang mit Gesundheitsanwendungen gewinnen und ihre Daten besser geschützt werden.
